# Effect of Lipopolysaccharide-Induced Apical Periodontitis in Diabetes Mellitus Rats on Periapical Inflammation

**DOI:** 10.1055/s-0042-1758790

**Published:** 2023-01-04

**Authors:** Eric Priyo Prasetyo, Galih Sampoerno, Devi Eka Juniarti, Febriastuti Cahyani, Widya Saraswati, Mefina Kuntjoro, Evelyn Tjendronegoro

**Affiliations:** 1 Department of Conservative Dentistry, Faculty of Dental Medicine, Universitas Airlangga, Surabaya, Indonesia; 2 Department of Prosthodontics, Faculty of Dental Medicine, Universitas Airlangga, Surabaya, Indonesia; 3 Healthcare and Research, Irvine Medical Center, University of California, Irvine, California, United States

**Keywords:** diabetes mellitus, lipopolysaccharide, apical periodontitis, inflammation, interleukin-6, tumor necrosis factor-α

## Abstract

**Objectives** To evaluate periapical inflammation through immunohistochemical analysis of interleukin 6 (IL-6) and tumor necrosis factor α (TNF-a) expression resulting from lipopolysaccharide (LPS)-induced apical periodontitis in diabetes mellitus rats, observed at 14, 28, and 42 days.

**Materials and Methods** Diabetes model on rats was induced by streptozotocin (STZ). Fifteen rats were injected with low-dose STZ for 5 days and waited for 5 days until the blood glucose level was stable and measured above 300 mg/dL confirmed by a digital glucometer. LPS was used to induce apical periodontitis. After performing access cavity, pulpal and root canal extirpation was done on the right mandibular first molar's root canal space of rats, under anesthesia. LPS of 1 mg/mL dose was induced in the pulpal and root canal space. Apical periodontitis was expected 14 days afterward and then, the rats were randomly allocated to three groups. The first group was terminated 14 days after induction and used as control. The second group was observed 28 days after induction, and the third group was observed 42 days after induction. IL-6 and TNF-a expression was analyzed by immunohistochemistry on macrophages in the periapical area.

**Statistical Analysis** Data were analyzed using one-way ANOVA and continued with the post hoc Tukey HSD test. Significance was considered if *p* < 0.05.

**Results** LPS induced apical periodontitis in diabetes mellitus rats at control (14 days), 28 and 42 days observation showed a significant increase in the expression of IL-6 and TNF-a. There were significant differences between the control and observed groups ( *p* < 0.05). The expression of IL-6 in the apical area was not significant at 14 and 28 days ( *p* > 0.05) but increased significantly at 42 days ( *p* < 0.05). The expression of TNF-a in the apical area was significantly increased after 14 days ( *p* < 0.05) and remained stable at 28 and 42 days ( *p* > 0.05).

**Conclusions** The periapical inflammation of LPS-induced apical periodontitis in diabetes mellitus rats increased macrophages' expression of IL-6 at 42 days and TNF-a at 28 days.

## Introduction

Diabetes mellitus is a worldwide issue affecting the young, adolescents, adults, and elderly. It is a chronic metabolic disease characterized by hyperglycemia caused by lack of insulin production, responsiveness, or both and the number of people suffering from this degenerative disease is expected to increase to more than 600 million world population by 2040. 1 Persistent and untreated hyperglycemia may lead to diabetic vascular complications involving macro and micro vasculatures through many structural and metabolic changes including in the oral tissues. 2 Poor metabolic control in hyperglycemia will increase the risk of dental complications in the oral cavity such as tooth decay, tooth loss, periodontal problems, pulp necrosis, and apical periodontitis. 3 4

Apical periodontitis is an inflammatory condition in the periapical area, causing tissue inflammation, destruction, and resorption. 5 This condition damages the tissues in the periapical area and involves systemic host factors including the production of inflammatory mediators. 6 Inflammatory mediators are closely related to involved microorganisms. One of many microorganisms responsible for periodontitis, periapical lesions, and endodontic treatment failures is *Porphyromonas gingivalis* . 7 8 *Porphyromonas gingivalis* is a gram-negative bacterium that has lipopolysaccharide (LPS) in its outer membrane. LPS is an endotoxin with a rigorous effect on periodontal tissue destruction and inflammation, including the induction of pro-inflammatory mediators. 9 10

Classic inflammatory mediators focused on in this study are tumor necrosis factor-α (TNF-a) and interleukin-6 (IL-6). IL-6 and TNF-a are important targets for an effective treatment strategy for their potency in periodontal destruction and erosion. 11 These mediators in hyperglycemic conditions are responsible for heightened levels of chronic inflammation, delayed periapical tissue healing and increased bone destruction. 12 IL-6 and TNF-a as pro-inflammatory cytokines can directly stimulate osteoclastogenesis, stimulate RANKL production, and act synergistically with RANKL to cause bone resorption. 13 In diabetes mellitus, the elevated amount of circulating IL-6 and TNF-a may impair glycemic control in association with endodontic inflammatory reaction. 14

Diabetes mellitus increases the severity and risk of inflammatory periodontal disease and both diseases have a bidirectional relationship. 15 Local infections in the oral cavity are also related to systemic conditions, target macrophages and neutrophils, and activate TNF-a, IL-6, other interleukins, α-interferon, prostaglandins, and the complement system. 16 In the process of apical periodontitis and bone loss in diabetic subjects, polymorphonuclear neutrophils and macrophages are prominent cells in the regulation of inflammation and bone destruction, including the secretion of pro-inflammatory cytokines leading to bone resorption. 17 Inflammatory profile of apical periodontitis is still not completely understood as the process is dynamic involving different inflammatory cells and their by-products which are interrelated. 18

Many studies use open-pulp methods to allow many bacteria to produce apical periodontitis, but this would lead to the vast destruction of rat teeth as teeth in subjects with diabetes mellitus are prone to decay. Previous studies have conducted various time range for observation from days to weeks. In this study, we investigated whether *P. gingivalis* LPS induction may produce apical periodontitis and evaluated the changes in IL-6 and TNF-a expression on macrophages after 14, 28, and 42 days in a diabetes mellitus rat model.

## Materials and Methods

### Animals

All experimental procedures were approved by the Health Ethics Commissions for Animal Studies, Faculty of Dental Medicine, Universitas Airlangga (approval number 040/2022). Fifteen healthy adult (10–12 weeks) male rats ( *Rattus norvegicus,* Wistar strain) weighing 250 to 300 g were used in this study. The animals were randomly divided into three groups with five rats each, with three experimental groups. The rats were cared for in diurnal lighting conditions (12 hours light/dark cycle) with a constant temperature of 25°C, and relative humidity of 45 to 55%. Rats have free access to food and water ad libitum.

### Diabetes Mellitus Model by Streptozotocin Induction

Accu-Chek Digital blood glucometer (Roche, Germany) was used to measure rats' blood glucose levels before, during, and 5 days after the last STZ injections. Streptozotocin (STZ) was purchased from Bio-World (USA). Diabetes mellitus induction by multiple low-dose STZ was according to a previously published method with modification on the dose of STZ and given glucose solution. 19

Rats were fasted for 6 hours before daily STZ low-dose injection intraperitoneally (20 mg/kg body weight) for 5 days consecutively. After every STZ injection, a 5% glucose solution (Otsuka, Indonesia) was given to the rats for 24 hours. Confirmation of successful diabetes model was done 1 week after STZ injection by measuring fasting blood glucose level of > 300 mg/dL. The blood glucose level of each rat was recorded.

### Apical Periodontitis Model by Lipopolysaccharide Induction

After diabetes mellitus induction on the rats was confirmed successful (blood glucose level > 300 mg/dL), LPS induction for the apical periodontitis model was performed in the following week. Ultrapure LPS from *P. gingivalis* was purchased (Invitrogen, USA) and mixing was done as per the manufacturer's instruction to obtain a concentration of 1 mg/mL.

Adult rats weighing 250 to 300 g were subjected to anesthesia with ketamine 40 mg/kg (Kepro B.V., The Netherlands) and xylazine 5 mg/kg (Xyla Interchemie, The Netherlands) by intraperitoneal injection. The involved right mandibular teeth and gingival areas were swabbed with Consepsis (Ultradent, USA). The mandibular right first molar was prepared using a dental handpiece (NSK, Japan) with round diamond bur no. 1/4 (Intensiv SA, Switzerland), access cavity, and pulpectomy were done using a barbed brooch (Dentsply, USA). Root canal preparation was done with K-file numbers 6, 8, 10, and 15 (Dentsply, USA). An electronic apical locator was used (Dentsply, USA) to assist with the preparation. Sterile saline (Otsuka, East Java, Indonesia) irrigation was done following root canal preparation using Navi-tips (Ultradent, USA). Root canals were dried with sterile paper point number 15 (Dentsply, USA). Prepared 1 mg/mL LPS was injected into the root canal space 25 microliters using a Hamilton microliter syringe (Hamilton, USA). The coronal was sealed with Cention glass ionomer (Ivoclar-Vivadent, Liechtenstein) to prevent contamination into the root canals.

The rats were terminated after 14 days, 28, and 42 days after induction of LPS by anesthetic overdose with ketamine 300 mg/kg and xylazine 30 mg/kg intraperitoneal injection according to the AVMA-recommended guidelines on euthanasia. The mandibles were dissected and immersed in 10% paraformaldehyde solution.

### Histopathological and Immunohistochemical Analyses

All samples were decalcified with a 5.0% aqueous solution of ethylene diamine tetra acetic acid (EDTA) for 90 days, embedded in paraffin blocks, and sliced by microtome at 5 µm thickness following standard histological protocol. Hematoxylin and eosin (HE) staining was used for histopathological examination. Sections containing treated teeth with apical periodontitis were focused on for consideration.

Immunohistochemical (IHC) staining was performed using monoclonal primary antibody anti-IL-6 (ABIN6002527, antibodies-online, Germany) and anti-TNF-a (ABIN3023860, antibodies-online, Germany) to assess macrophage expression. Macrophages were counted in the periapical region. Histopathological and immunohistochemical specimens were analyzed microscopically using a light microscope Nikon Eclipse Ci-E (Nikon Corp, Japan) focusing in the periapical area of apical periodontitis. Expression of IL-6 and TNF-a of macrophages was counted from IHC-stained specimens at 400× magnification.

### Data Analysis

All data were computed and are expressed as mean ± standard deviation. Statistical analysis was processed for normality using the Shapiro–Wilk test and homogeneity using the Levene test. Data were evaluated using a one-way analysis of variance (ANOVA) test followed by a Tukey honest significant difference (HSD) test between the groups. A *p-* value of less than 0.05 was considered statistically significant.

## Results

### Blood Glucose Level

Before STZ treatment, the blood glucose level of each rat was measured as normal (70–130 mg/dL). During STZ treatment, the blood glucose level rises variably (140–310 mg/dL), and after the completion of 5 days of injections, the blood glucose level was stable at 300 to 490 mg/dL. The level remains stable after 5 days from the last injection. All 15 animals survived multiple low doses of given STZ induction.

### Apical Periodontitis Induction

**Fig. 1:**
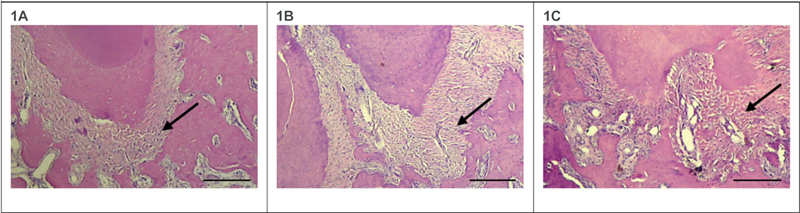
Histopathological examination in the apical area (indicated by black arrows) of LPS-induced AP in DM rats. Scale bar: 200 μm. **1A** showing apical periodontitis at control, **1B** showing apical periodontitis at 28 days of observation, and **1C** showing apical periodontitis at 42 days of observation. Compared with the control group, apical periodontitis at 28 days and 42 days were larger in size. The structure of apical periodontal tissues at 42 days showed the most irregularity.

Ultrapure *P. gingivalis* LPS induction resulted in the formation of apical periodontitis in the periapical area. The histopathological examination of induced apical periodontitis can be seen in Fig. 1 . The images were obtained from a light microscope (Nikon Eclipse Ci-E, Japan) with 40× magnification.

### Inflammation in the Apical Area

**Fig. 2:**
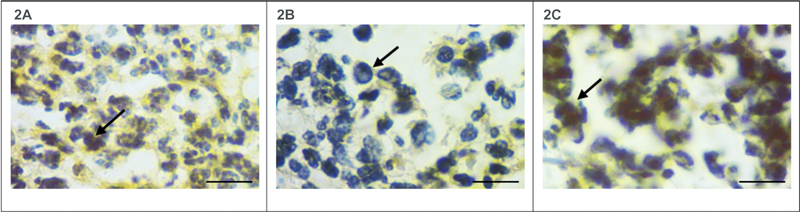
Immunohistochemistry examination of IL-6 expression on macrophages (indicated by black arrows) in the apical area of LPS-induced AP in DM rats. **2A** showing the expression of IL-6 at control, **2B** showing the expression of IL-6 at 28 days of observation, and **2C** showing the expression of IL-6 at 42 days of observation.

**Fig. 3:**
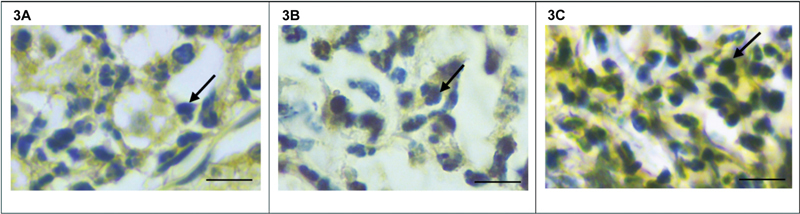
Immunohistochemistry examination of TNF-a expression on macrophages (indicated by black arrows) in the apical area of LPS-induced AP in DM rats. **3A** showing the expression of TNF-a at control, **3B** showing the expression of TNF-a at 28 days of observation, **3C** showing the expression of TNF-a at 42 days of observation.

Expression of IL-6 and TNF-a on macrophages in the periapical area was analyzed by immunohistochemical staining ( Figs. 2 and 3 ). The images were obtained from a light microscope (Nikon Eclipse Ci-E, Japan) with 400× magnification. Inflammatory cells were found around the periapical area. The comparison of IL-6 and TNF-a expression between groups can be seen in Tables 1 and 2 . Significances were found in both groups of IL-6 and TNF-a. IL-6 expression was significantly increased at 42 days, while TNF-a expression was significantly increased at 28 days and remained stable at 42 days compared with the control group.

**Table 1:** Mean and standard deviation of IL-6 and TNF-a expressions among groups

Groups	*n*	IL-6 expression (mean ± SD)		TNF-a expression (mean ± SD)	
Control	5	5.800 ± 1.114	*p* = 0.034*	6.160 ± 1.161	*p* = 0.001*
28 days	5	6.920 ± 0.610		8.880 ± 0.867	
42 days	5	7.960 ± 1.493		9.320 ± 0.996	

* Significant difference.

**Table 2:** Significance of IL-6 and TNF-a expression among groups

**Comparisons**	**IL-6 expression** **(** ***p*** **-value)**	**TNF-a expression** **(** ***p*** **-value)**
Control to 28 days	*p* = 0.297	*p* = 0.003*
Control to 42 days	*p* = 0.027*	*p* = 0.001*
28 days to 42 days	*p* = 0.346	*p* = 0.776

* Significant difference.

## Discussion

The model to evaluate diabetes mellitus and apical periodontitis in rats is well established. 19 STZ is the agent of choice for reproducible diabetes mellitus induction for an animal model, and the metabolism resulting in rats is very similar to the metabolism in humans. 20 Previous studies have shown that the process of periapical tissue is similar to that observed in humans. Root development is complete in 16 weeks old rat molars, and the similarity of histological features in rat teeth benefits the use of rats in our study. Several studies have experimented the induced apical periodontitis by pulp exposure to infection from oral microorganisms. 21

Our study provided a detailed model of using LPS to induce apical periodontitis in diabetes mellitus rats. LPS, an endotoxin of *P. gingivalis* , gram-negative bacteria, when in contact with host cells induces an immune-inflammatory reaction, at a certain concentration would develop tissue damage. 22 In this study, access opening and closed induction of LPS was chosen to preserve tooth structure, as the open method will result in faster tooth decay and deteriorate tooth structure, especially in the diabetes mellitus model in which the tooth structure is prone to decay and have high caries risk. 1

LPS as endotoxin binds to Toll-like receptors 4 (TLR-4) in the host's innate immune system and activates nuclear factor kappa B (NFKB) to trigger the release of pro-inflammatory cytokines. 16 Inflammatory conditions in the periapical region can be determined by the expression of IL-6 and TNF-a. As dominant inflammatory cells in chronic periodontitis, macrophages (M1) express and secrete a range of proinflammatory mediators such as IL-6 and TNF-a, which reduce insulin signaling and contribute to insulin resistance and affect the balance between M1 and M2 macrophages to determine the inflammatory effect. 23 Previous studies have demonstrated that macrophages abundantly play a crucial role in apical periodontitis development involving their activation and migration. 24

Diabetes condition increases inflammation, this is in accordance with our study, which showed an increase in the expression of IL-6 and TNF-a in inflammatory cells. This inflammation is suggested to be triggered by oxidative stress, including the increased levels of advanced glycation end-products (AGEs) as a result of prolonged hyperglycemia, and eventually further activate pro-inflammatory pathways and may cause epigenetic changes of persistent expression of proinflammatory genes even after the hyperglycemia is controlled. 23 In this case, epigenetic involvement is related to the persistence of apical periodontitis.

IL-6 is synthesized at low levels in healthy periapical tissues but might be drastically upregulated during periapical inflammation. 25 High expression of IL-6 in apical periodontitis is correlated with active symptomatic lesions, but the relation to systemic inflammatory condition showed conflicting results. 18 IL-6 is a key cytokine expressed and released by several inflammatory cells, including macrophages, associated with bone resorption and host defenses. 26 Higher IL-6 level is positively associated with the size of the lesion, while higher TNF-a level is associated with exudation. 27

In our study, IL-6 expression is gradually increased and statistically significant between control and day 42, indicating greater macrophage presence in the periapical area over a longer time, but the increase is not significant between control to 28 days and 28 to 42 days. This showed the diabetes mellitus condition delayed the healing process of apical periodontitis resulting from closed induction of LPS. IL-6 is important in immune responses, expressed during inflammation, after TNF-a to play a protective role in immune reactions and bone resorption. 28 IL-6 is induced by TNF-a and IL-1 during the early stages of inflammation-promoting inflammatory cell recruitment, moving from acute to chronic stage, inducing the expression of matrix metalloproteinase, and stimulating osteoclast differentiation and bone resorption. 25 Other possible factors to consider about the increase of IL-6 at 42 days and TNF-a at 28 days are the interaction or homeostasis of cytokines and chemokines during periodontitis state, 29 such as IL-1a, IL-1b, IL-1ra, IL-4, IL-10, IL-12, and IL-17, and this process could be imbalanced or disturbed in diabetes mellitus condition. In humans, higher levels of IL-6 were significantly found in symptomatic apical periodontitis compared with asymptomatic apical periodontitis, representing an active process immunologically. 13

TNF-a is strongly correlated to the incidence and exacerbation of periodontitis in diabetes mellitus. 30 TNF-a is a major cytokine released by activated macrophages, monocytes, and T lymphocytes which affect growth regulation, immune response, survival, differentiation, and physiological functions of different cells together with the production of other enzymes, mediators, and cytokines. TNF-a production to increase disease progression is triggered by endotoxins. 31 The role of TNF-a expression on bone resorption and exacerbation is established in apical periodontitis. 21 TNF-a is a potent inducer of bone resorption among other cytokines in apical periodontitis. 32 TNF-a interferes with autophagy and modulates pro-inflammatory responses, cell differentiation, proliferation, and death. 31 TNF-a expression is correlated with the amount of macrophages in the surrounding tissues, indicating the production of this cytokine and inflammation status. 18

TNF-a is expressed from day 7 after pulpal exposure and from days 28 onward, while a positive correlation is found between TNF-a levels and destruction size in rats. 21 Complete healing in the peri-radicular tissue was not resolved in this study. Diabetes mellitus condition remaining in the systemic milieu enables apical periodontitis to persist. This study did not include root canal treatment to study the condition of periapical destruction. The present study showed the presence of periodontal destruction and lower bone density in the periapical region. The constant number of inflammatory cells was related to diabetes mellitus where continuous hyperglycemic condition plays a crucial part in the tissue healing modulation process.

Patients with diabetes and inflammatory stimulation of LPS showed higher levels of IL-6 and TNF-a, and IL-6 levels are strongly correlated with the severity of disease progression. 16 In line with our study results, clinical studies reported significantly higher expression of IL-6 and TNF-a synergistically in periapical lesion progression and stability to stimulate bone resorption and osteoclast differentiation via RANK/RANKL/OPG mechanisms. 33 34 The pattern of tissue destruction in the periapical area of rats over control, 28, and 42 days observation showed diabetes mellitus with hyperglycemia as a stimulus for healing difficulties, increased risk of infection, and reduced recovery is in agreement with our result. Histological observation showed necrotic periapical areas with irregular apical periodontal tissue structure.

Periapical tissue repair is a coordinated process involving highly related events, including cell proliferation and migration, growth factors, and cytokine release. In the mechanism of apical periodontitis, cytokines are involved, including IL-6 and TNF-a for bone resorption, osteoclast activity, and proinflammatory cytokines. Immune and systemic systems interact with one another, sharing fundamental interactions that are involved in the pathophysiology of periodontal tissues, including apical periodontitis. Previous studies presented that the same bacterial stimulus-induced greater inflammation in diabetic animals compared with normal control, and diabetes significantly increase the severity and susceptibility of periodontal disease. 35 Other factors such as stress oxidative parameters are partly responsible for the higher and more aggressive apical periodontitis in diabetes mellitus. 36 37 Further studies should be done to investigate the effects on other inflammatory mechanisms and therapeutic effects. Understanding inflammation is expected to set a basis for further study on endodontic regeneration and treatments in the apical area for subjects with diabetes mellitus.

## Conclusion

Our study demonstrated that LPS from *P. gingivalis* can produce apical periodontitis in diabetes mellitus rats in an *in vivo* model, and affect the expression of IL-6 and TNF-a. Observation times of 28 and 42 days influence the inflammatory status of apical periodontitis, especially in diabetes mellitus condition. The periapical inflammation of LPS-induced apical periodontitis in diabetes mellitus rats increased macrophages' expression of IL-6 at 42 days and TNF-a at 28 days. Further studies are important to understand the mechanism of time, diabetes mellitus, apical periodontitis, and other contributing factors, for endodontic treatment and regeneration in the apical area, especially in subjects with diabetes mellitus.
